# Are metal-based antibacterial gels a potential alternative for disinfection in contemporary endodontics?

**DOI:** 10.1038/s41432-024-01102-7

**Published:** 2025-01-07

**Authors:** Bryan D. Murchie, David Edwards

**Affiliations:** 1 Clinical Lecturer in Restorative Dentistry, School of Dental Sciences, Faculty of Medical Sciences, Newcastle University, Newcastle Upon Tyne, UK; 2 Doctoral Research Fellow and Specialty Trainee (Endodontics), School of Dental Sciences, Faculty of Medical Sciences, Newcastle University, Newcastle Upon Tyne, UK

**Keywords:** Root canal treatment, Pulpitis

## Abstract

**Aims:**

This study aimed to assess the effectiveness of a novel antimicrobial gel, containing copper and silver nanoparticles, for use in root canal disinfection.

**Methods:**

Copper and silver-based gels were created in-house, using a support network of biocompatible polymers, including polyvinyl alcohol (PVA), polyvinyl pyrrolidone (PVP), and polyethylene glycol (PEG). Six experimental groups were created, three containing silver ions and three copper ions, where the PVA, PVP and PEG ratios were also adjusted in each group to test the gel’s physical state. One control contained no metal nanoparticles. The gels surface characteristics, roughness, mechanical properties, and flowability, were characterised using a combination of atomic force microscopy, scanning electron microscopy, transmission electron microscopy and Rheometery. Further biological testing measured the gels cytotoxicity levels, using human periodontal ligament cells, and the anti-microbial effects against *E. Faecalis* and a multi-species bacteria biofilm.

**Results:**

Each gel demonstrated high levels of viscosity, which was lowered in gels containing a reduced PVA concentration. The overall antimicrobial properties of the gels increased in those with a higher dissolution, lower porosity, and reduced surface roughness. Copper nanoparticles were shown to be significantly more effective against *E. Faecalis*, compared with silver. Gels containing higher PVA levels, and silver nanoparticles, had greater toxicity levels against human cells, however, testing was not possible for most experimental groups as the gels dissolved before measurements took place. The antimicrobial properties of all gel formulations were significantly less effective than sodium hypochlorite (after 1 h), but a similar outcome was detected in comparison with calcium hydroxide (after 7 days).

**Conclusions:**

Developing an antimicrobial gel is highly dependent upon numerous compositional factors, where development is still at the early stages. The use of copper nanoparticles appeared to be more appropriate for use in canal disinfection, compared with silver that also had higher levels of human cell toxicity. The ratios selected for the biocompatible polymers had a critical impact on the physical state, antimicrobial, and toxicity levels. At present, antimicrobial gels are not as effective as sodium hypochlorite.

## A Commentary on

**Berri M E, Jerez-Olate C, Ramírez J A** **et al**.

Novel Antibacterial and Biocompatible Nanostructured Gels Based on One-step Synthesis as a Potential Disinfectant for Endodontic Infection Control. *J Endod* 2024; **50:** 74–84.

**GRADE Rating**: 

## Commentary

Effective chemomechanical disinfection lies at the core of endodontic principles for success. The complex anatomy of the root canal system, within which microbial infection exists in a biofilm, means the chemical component of this approach is critical^1,2^. Common antimicrobial irrigant solutions available on the market today include sodium hypochlorite (NaOCl), citric acid, EDTA, and chlorohexidine. These may be supplemented through interappointment dressings, such as calcium hydroxide (CH), where the bactericidal medicament is left in between visits. At present, there is no ‘ideal’ method that can completely and safely disinfect the root canal system, with eradication of all microorganisms and dissolution of necrotic organic debris^3^. The limitations of existing disinfectants and dressings has driven research into seeking for alternative methods. Although NaOCl remains the gold standard irrigant, recent publications have identified the potential effectiveness of metal nanoparticles, due to their inherent antimicrobial properties. However, the applications, and use of, different metals in endodontics have not been thoroughly explored to-date^4^.

The introduction of this paper^5^ demonstrated compelling arguments in favour of introducing a new method for achieving intracanal disinfection, with consideration of contemporary treatments. Interestingly, the authors did not commit their novel gel to use as either an irrigant solution, or interappointment dressing, highlighting the general levels of uncertainty around the clinical application of these gels.

In total, seven different in vitro methods were used in this study, including: atomic force microscopy (AFM), scanning electron microscopy (SEM), transmission electron microscopy (TEM), energy dispersive x-ray spectroscopy (EDS), Rheometery, confocal microscopy, and fluorescence microscopy. Methods were not always clearly aligned with objectives of the study, with the relevance of some methods to the overall aim being unclear.

As part of the study design, the control group utilised polymers without metal nanoparticles. However, it may have been even more informative to include other well-established methods of chemical disinfection, such as NaOCl and/or CH. This would have increased the external validity of the study, considering they are already an established part of clinical practice. It was noted that both products were included in the later sections, when investigating the anti-bacterial properties. However, the authors did not make it clear why these products were not universally tested in all experiments.

The results of all experiments were mostly reported, including detailed tables and high-quality pictures that were well presented and easy to interpret. One of the in vitro methods (EDS) was mentioned earlier in the text under the methods section, but then the results were not presented. Much of the data were qualitative, where the findings should be interpreted with caution as blinding did not occur, risking operator bias. It may have been possible to obtain more quantitative data in some experiments. For example, the AFM is well-known to provide mechanical information at nanoscale, which would have been an ideal method design for this experiment to test the gels modulus of elasticity, viscoelastic, and/or hardness properties. It was noted that a rheometer was used to test the fluid dynamics and mechanical properties, which would have been interesting to compare with the AFM findings.

The results section highlighted that cytotoxicity testing methods were not compatible with most gel compositions, which dissolved upon contact with the cell cultures. Antimicrobial comparisons with sodium hypochlorite demonstrated that the gels were significantly less effective against *E. Faecalis*, after short a 1-h exposure, whereas longer exposures (7-days) had a comparable outcome with CH. Direct comparisons were not possible as each product was tested differently, the reasons of which are unclear.

A limitation of this study is the lack of comparison of most experiments to a control group. Only the roughness levels, antibacterial and cytotoxicity levels were compared to the experimental gel groups. This reinforced the need to justify each testing method in line with the aims of the study.

The discussion explored all the most relevant findings, with reference to the original aim of the paper. The authors explored ways to improve their current gel formulations for future research. In particular, it was clear that the gel was highly viscous, which is not an ideal property for disinfection considering the complex anatomy of root canal systems and the ability of bacteria to penetrate dentinal tubules. It was discussed that a successful balance of minimising cytotoxicity whilst maintaining anti-microbial properties had been achieved in this experiment. However, the results reported that the toxicity levels could not be confirmed for the majority of experimental groups. Furthermore, NaOH was significantly more effective against *E. Faecalis* than all gel formulations. Although, limited findings showed that copper-based gel appeared to be more effective in both areas compared to silver ions. Therefore, it could be argued that the aim for this study was broadly met. Authors also acknowledged what they considered as the main limitation of this experiment, where they only tested each gel against a single bacterial species (*E. Faecalis*). Although, this was inherent issue associated with the laboratory-based design, which cannot mimic all relevant *in vivo* environmental conditions.

## Limitations

In addition to the above comments, a further limitation was the exclusion of either human teeth, or standardised 3 d models of human root canal systems. AFM could have also tested for mechanical changes to the root dentine post-exposure, as this was a key factor not considered in this study, and essential to the long-term prognosis. It is widely reported that irrigants and dressings may damage dentine, increasing the risk of fracture and impacting sealing of the root canal system.

## Summary

This study presents an alternative approach for managing root canal disinfection utilising a novel antibacterial gel, infused with metal nanoparticles (silver or copper). Although, this study has highlighted that development is still in the very early stages, and much additional research is needed to ensure that this is a safe and effective alternative to current practices. It is unclear what role exactly these gels would form as part of the endodontic armamentarium, e.g., irrigant or medicament between visits. This question is something that would be addressed at a later stage once other aspects have become clear, such as the flowability and the effects on dentine’s mechanical properties.

Practice points

- This study shows that antimicrobial gels cannot currently be recommended to supplement or replace current practice.
- Antimicrobial gels show future prospect but are still in the early stages of development.
